# Revolution of Circulating Tumor DNA: From Bench Innovations to Bedside Implementations

**DOI:** 10.3390/cimb47060428

**Published:** 2025-06-06

**Authors:** Xuehan Yan, Juncheng Su, Zheng Wang

**Affiliations:** Department of Gastrointestinal Surgery, Renji Hospital Affiliated to Shanghai Jiao Tong University School of Medicine, Shanghai 200127, China; dr_yanxh@126.com (X.Y.); suoxiansjc666sjtu888@sjtu.edu.cn (J.S.)

**Keywords:** ctDNA, cancer, detection method, clinical application

## Abstract

Circulating tumor DNA (ctDNA), a newly developed cancer biomarker, consists of single- or double-stranded DNA fragments that are shed from tumor cells in primary or metastatic sites. They are released into peripheral blood and exhibit distinct characteristics associated with cancer, even in the early stages. With advancements in technology, ctDNA detection has become more diverse and precise, including digital Polymerase Chain Reaction (dPCR) and next-generation sequencing (NGS), among others. As a chronic disease that develops over an extended period, early detection is crucial for the accurate diagnosis and treatment of cancer and can significantly improve patient prognosis. Therefore, analyzing ctDNA features is important. Additionally, ctDNA can be used to assess post-surgical minimal residual disease (MRD), aiding in treatment decisions. Overall, ctDNA plays a crucial role in the progression of cancer and its treatment. This review summarizes the sources and features of ctDNA, the analytical techniques used, and its application in both solid and non-solid tumors.

## 1. Introduction

**Figure 1:**
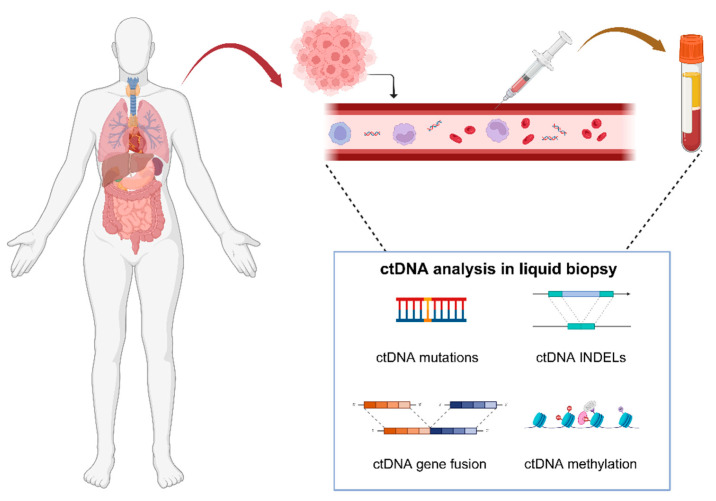
Graphic abstract of ctDNA analysis. The bloodstream contains tumor-derived circulating tumor cells, extracellular vesicles, and free nucleic acids. By collecting blood samples from cancer patients and isolating ctDNA, we can analyze its characteristics, including mutations, amplifications, deletions, gene fusions, and epigenetic modifications such as methylation.

The introduction of “The Hallmarks of Cancer” theory by Douglas Hanahan and Robert A. Weinberg in 2000 has made biomarker research a prominent focus in oncology over the past two decades [1]. Over time, new theories on tumor biomarkers have emerged, such as genome instability and mutation [2], as well as nonmutational epigenetic reprogramming [3]. DNA is an essential genetic information repository that plays a crucial role in guiding cellular activities, and the analysis of it offers a comprehensive understanding of cellular characteristics. Traditionally, DNA was believed to reside within the nucleus; however, in 1948, Mandel and Metais first discovered DNA in human plasma, which is termed cell-free DNA (cfDNA) [4]. cfDNA refers to DNA released from cells into the circulating plasma, with the DNA derived from tumors called circulating tumor DNA (ctDNA) [5]. Alterations in DNA can profoundly influence cellular activities and may promote their malignant transformation. Deletions, duplications, frameshift mutations, and point mutations can change DNA sequences [6,7], while methylation can significantly impact the expression of the genetic information encoded in DNA [8,9]. Consequently, liquid biopsy for ctDNA has been developed [10,11]. Extracting and analyzing DNA fragments from peripheral plasma allows us to gather insights into the diverse cellular activity states [10,11] (Figure 1).

In clinical practice, common methods for detecting ctDNA include digital Polymerase Chain Reaction (dPCR) and next-generation sequencing (NGS) [12,13,14,15]. dPCR divides the traditional PCR reaction system into numerous small systems, each containing only one copy of the molecule for amplification and quantification [12,13]. In contrast, NGS enables the parallel sequencing of a large number of DNA molecules [15]. Both dPCR and NGS sequencing possess distinct advantages and limitations, so the selection of the method should be guided by the specific clinical context in clinical practice.

Early screening is essential in the clinical progression of tumors, where ctDNA is an efficient tool [16]. It allows clinicians to intervene before tumor invasion or metastasis occurs, thereby significantly improving a patient’s survival probability [17,18]. Although postoperative minimal (or molecular) residual disease is routinely checked after surgery to confirm a complete excision of the tumor, while regular CT/PET-CT scans are used to detect metastasis, ctDNA can identify tumor recurrence at the molecular level before metastases are detectable in imaging, thus aiding in the assessment of treatment efficacy [19,20,21].

ctDNA holds substantial value for early screening and therapeutic assessment, and in this review, we will start with the origins and characteristics of ctDNA by comparing it with cfDNA to highlight its specificity. Next, we will compare dPCR and NGS, the two most widely used techniques for ctDNA detection, and identify their optimal application environments. Finally, we will summarize the clinical applications of ctDNA across different cancers by reviewing the existing literature.

## 2. General Description of ctDNA

### 2.1. Origins

ctDNA, a subset of cfDNA, shares the same origins as cfDNA (Table 1). The sources of cfDNA primarily involve two mechanisms: passive release, occurring after cellular breakdown, and active release, involving the deliberate secretion of DNA by cells [22]. Passive release mainly originates from cells that are injured, dying, or dead, including those undergoing apoptosis, necrosis, pyroptosis, or autophagy, while active DNA release occurs via the active secretion of particular structures [22,23,24]. In cancer patients, cfDNA originates from healthy cells, malignant cells, and cells within the tumor microenvironment [23]. The DNA released by malignant and tumor microenvironment cells carries tumor-specific genetic features and is collectively known as ctDNA [25]. The tumor microenvironment comprises tumor cells, stromal cells, and various immune cells, all of which may contribute to ctDNA [22,23]. Additionally, circulating tumor cells (CTCs) and exosomes also serve as sources of ctDNA, as they originate from primary tumors or metastases, penetrate the vascular wall, and enter the bloodstream [26,27,28] (Figure 1).

### 2.2. Features

Although ctDNA is a subset of cfDNA, it possesses unique characteristics, as summarized in Table 1. Unlike cfDNA, which is present in all individuals, ctDNA is found exclusively in cancer patients, derived from tumor-associated cells, and carries specific genetic features, such as gene mutations and methylation, which serve as markers for malignant cells [29,30,31]. In addition, ctDNA and cfDNA exhibit significant differences in molecular length. Due to apoptosis, the short DNA fragments reflect the underlying histone octamer structure, whereas necrotic cells release longer fragments [32]. Typically, apoptotic processes generate DNA fragments of around 166 bp, comprising 147 bp that are wrapped around a nucleosome, plus additional DNA that is associated with histone H1. Furthermore, depending on the nuclease activity, apoptosis may yield even longer fragments spanning multiple nucleosomes [32]. In contrast, a significant portion of tumor-derived cfDNA is highly fragmented, with fragment sizes below 100 bp [33]. The concentration of cfDNA is positively correlated with the tumor burden [23]. Previous studies on breast, colorectal, and prostate cancers have shown that plasma cfDNA levels in cancer patients are significantly higher than those in healthy controls, with an approximately 3- to 30-fold increase [34,35,36,37,38,39]. Moreover, patients with metastatic tumors exhibit even higher cfDNA levels than those with primary tumors [40,41]. Despite the elevated cellular proliferation, enhanced apoptosis, and tumor-associated immune activation that collectively generate a disordered microenvironment conducive to DNA release [1,2,3], circulating tumor DNA (ctDNA) constitutes merely a minor fraction (typically <1–10%) of the total cell-free DNA (cfDNA) in most malignancies [20]. Further research is needed to understand why cfDNA levels are significantly elevated in the plasma of cancer patients.

### 2.3. Clinical Applications

As a non-invasive test, liquid biopsy based on cfDNA and ctDNA has been applied in clinical practice and proven valid [42,43]. According to its specificity mentioned above, ctDNA is primarily associated with tumor-related clinical applications, including early screening [44], detection of postoperative MRD [19,20], recurrence monitoring [45], and therapeutic assessment [46]. These aspects will be discussed in detail in the following sections. In contrast, cfDNA has broader applications, as it reflects the overall cellular state in the body, which plays an important role in prenatal screening [47], organ transplant monitoring (such as the liver [48] and kidney [49]), and the diagnosis of chronic diseases [50]. However, the applications of cfDNA are beyond the scope of this review.

## 3. Detecting Techniques for ctDNA

### 3.1. dPCRS

Digital PCR is an absolute quantitative nucleic acid detection technology based on a single-molecule PCR method [51]. Currently, dPCR is mainly classified into Droplet Digital PCR (ddPCR) and Chip Digital PCR (cdPCR), with ddPCR being more widely used [52,53]. The term “digital” describes this PCR technique due to its binary “all-or-none” characteristic, meaning each unit either contains no target molecules or a single molecule [54]. Compared to conventional PCR, dPCR offers several advantages. Firstly, dPCR does not rely on Ct values or reference genes, allowing for an absolute quantification of nucleic acid samples with single-copy sensitivity. Secondly, dPCR exhibits extremely high mutation detection sensitivity, which is capable of identifying a single mutant molecule among 100,000 wild-type molecules, outperforming ARMS–PCR and Clamping PCR. Additionally, because dPCR physically partitions the reaction components, it demonstrates a greater tolerance to PCR inhibitors, reducing the impact of background noise on detection accuracy [55]. Moreover, compared to traditional PCR, which typically detects mutant allele frequencies (MAFs) above 10%, dPCR significantly improves detection sensitivity, with ddPCR capable of detecting MAFs as low as 0.001% [56].

### 3.2. NGS

Next-generation sequencing is a high-throughput DNA sequencing technology that is capable of sequencing millions of DNA molecules simultaneously [57]. The NGS workflow comprises three key steps: (1) library preparation, where DNA fragmentation and adapter ligation enable platform attachment, primer binding, and multiplex sequencing [57,58]; (2) amplification, using bridge PCR (Illumina platforms) or emulsion PCR (bead-based systems) to generate clonal clusters [55,57,59]; (3) sequencing-by-synthesis with fluorescently labeled nucleotides for base detection, followed by data analysis involving quality control, reference genome alignment, and the identification of SNPs (Single-Nucleotide Polymorphisms), Indels (insertions and deletions), CNVs (Copy Number Variations), and SVs (Structural Variations).

### 3.3. Comparisons

As two of the most common methods used for detecting ctDNA in clinical practice, we list the differences between dPCR and NGS in Table 2. In terms of sample preparation, dPCR does not require a large amount of sample; even a very small quantity is sufficient for detection, while NGS has very high requirements for both sample quality and quantity. dPCR relies on highly specific primers for amplification and detection, which limits its application to the targeted detection of known mutation sites. Typically, the primers used in dPCR are designed for specific mutation sequences, such as common single-nucleotide mutations like KRAS^G12/G13^ and BRAF^V600^ [60,61]. These primers efficiently recognize and amplify the intended mutation, enabling highly sensitive and precise quantification. However, since dPCR can only detect pre-specified mutation sites, it fails to identify unexpected or rare mutations if they occur at different locations. In addition, some pathogens with known sequences can also be detected using dPCR technology [62]. For point mutations, dPCR offers higher precision than NGS; however, it is not suitable for the detection of unknown mutations or more complex genetic alterations, such as insertions, deletions, and gene fusions, where NGS is required [57]. Moreover, dPCR requires less time and effort compared to NGS, making it more convenient for rapid and targeted analyses [63].

When it comes to the detection of ctDNA, the simplicity, convenience, and high sensitivity of dPCR make it the method of choice if common mutations are suspected. Conversely, when the dPCR results are negative, NGS offers clinicians a more comprehensive genomic overview, enabling the detection of rare and complex genetic alterations [12,14,57]. In clinical practice, dPCR is generally preferred for detecting known low-frequency mutations (e.g., single-driver mutations such as KRAS^G12/G13^ and BRAF^V600^ or single amplification like HER2), high-sensitivity quantification scenarios (e.g., MRD monitoring, or postoperative or post-therapy ctDNA tracking), and limited-sample contexts (e.g., cerebrospinal fluid ctDNA analysis) [51,54,56,60,61]. Conversely, NGS is more commonly utilized for pan-cancer screening with multi-gene panels (when predefined mutation targets are unavailable), tumor heterogeneity profiling through subclonal variant detection, immunotherapy biomarker evaluation (including tumor mutational burden and a microsatellite instability assessment), and the exploration of unknown resistance mechanisms involving emerging mutations or fusion genes [56,57,58].

## 4. Applications of ctDNA in Clinical Practice

In 2017, Lim et al. provided a comprehensive overview of the clinical applications of ctDNA [64]. However, with significant updates in detection technologies and more implementations of ctDNA-related clinical trials, this section places greater emphasis on the clinical studies published within the past ten years. We systematically aggregated and analyzed the research focusing on pan-cancers or the five most prevalent malignancies worldwide, which are ranked by incidence as follows: lung cancer, breast cancer, colorectal cancer, prostate cancer, and gastric cancer [65], aiming to provide additional perspectives.

### 4.1. Early Screening

Early screening is essential for improving the overall prognosis of cancer patients. It is widely acknowledged that detecting tumors at an early stage and performing timely surgical intervention may enhance cancer cure rates [66,67,68]. ctDNA, as a non-invasive diagnostic method, demonstrates significant advantages in cancer diagnosis and screening. Firstly, for tumors such as pancreatic cancer and ovarian cancer, which are often diagnosed at advanced stages due to the absence of symptoms and the lack of specific serum biomarkers [69,70], the application of ctDNA in early screening holds promise for detecting tumors at earlier developmental stages [40], thereby facilitating early diagnosis and treatment. Secondly, for highly heterogeneous tumors such as colorectal cancer, ctDNA provides a more homogeneous and comprehensive assessment of tumor classification compared to a direct tissue biopsy, which often involves sampling small tissue fragments. It is generally accepted that the development and validation of a qualified early screening or diagnostic method should be achieved through three distinct steps: (1) Analytical validation: The ability of the assay to accurately measure the analyte of interest, which has been described in the front part. (2) Clinical validation: The ability of the assay to measure the clinical feature of interest reliably and accurately. (3) Clinical utility: There is evidence of improved clinical outcomes compared with the standard methods [10].

The detection systems for ctDNA in early screening primarily include three methods: (1) mutation-based detection, (2) methylation-based detection, and (3) DNA fragment length-based detection. For mutation-based detection, Phallen et al. first utilized a highly sensitive detection method known as targeted error correction sequencing (TEC-Seq), revealing significant differences in the MAFs between cancer patients and healthy individuals [71]. Subsequently, CancerSEEK integrated ctDNA mutations with protein biomarkers to enhance screening sensitivity. This approach analyzed eight protein biomarkers and mutations in 16 genes, utilizing machine learning algorithms to predict the presence of cancer and its tissue of origin, which becomes the main method for mutation-based detection in early screening [72]. For methylation-based detection, the GRAIL test (also known as the Galleri test) is one of the most prominent plasma methylation tests for early cancer detection. Since methylated nucleotides in ctDNA are more abundant than mutated nucleotides and demonstrate stronger tissue specificity, methylation-based ctDNA detection offers higher sensitivity and specificity compared to single-gene mutation detection and is therefore widely used in multicancer early detection. For fragment length-based detection, studies have shown that the peak length between cfDNA and ctDNA is different. Then, Cristiano et al. utilized low-coverage whole-genome sequencing and machine learning to analyze the differences in DNA fragment profiles between healthy individuals and cancer patients, leading to the development of an early cancer diagnostic tool called DELFI (DNA Evaluation of Fragments for Early Interception) [73]. While DELFI exhibits higher sensitivity compared to CancerSEEK and GRAIL, its specificity is relatively lower. Additionally, the method has limitations in accurately determining the tissue of origin of ctDNA. Due to its high sensitivity, further research has focused on optimizing DELFI analysis and expanding its applications.

For each detection method, both completed and ongoing clinical studies are comprehensively summarized in Table 3, and some of them are described in detail below, all of which were obtained prior to treatment initiation.

The first study using the CancerSEEK method evaluated the ctDNA levels in 1005 cancer patients (covering ovarian, liver, gastric, pancreatic, esophageal, colorectal, lung, and breast cancers) and 812 healthy individuals. The results showed a median sensitivity of 70% across the eight cancer types, with the highest sensitivity observed in ovarian cancer (98%) and the lowest in breast cancer (33%), while specificity exceeded 99%. For tumors at stages I, II, and III, the average sensitivities were 43%, 73%, and 78%, respectively [74]. DETECT-A (Detecting cancers Earlier Through Elective mutation-based blood Collection and Testing) is a subsequent prospective clinical trial of CancerSEEK, designed to evaluate its practical application in a larger asymptomatic population [75]. The study enrolled 10,006 asymptomatic women aged 65–75 years who underwent two rounds of blood testing using the CancerSEEK method. Confirmatory testing rigorously excluded the false positives caused by clonal hematopoiesis of indeterminate potential (CHIP). Patients with positive CancerSEEK results were further evaluated using PET-CT to confirm tumors, and the screening efficacy of combining PET-CT was assessed. All participants were followed for 12 months to monitor cancer occurrence. The results demonstrated a sensitivity of 27.1%, specificity of 98.9%, and positive predictive value (PPV) of 19.4% for CancerSEEK. When combined with PET-CT, the sensitivity decreased to 15.6%, the specificity increased to 99.6%, and the PPV improved to 28.3%.

For methylation-based detection, the GRAIL test employs targeted whole-genome bisulfite sequencing of plasma DNA and machine learning to analyze a panel of over 100,000 informative methylation sites. The CCGA (Circulating Cell-free Genome Atlas) study (NCT02889978) is the first prospective case–control clinical trial to explore the application of the GRAIL test in early screening. It is divided into three substudies: discovery analysis, initial validation, and large-scale clinical validation. In the first substudy, the study compared the clinical limit of detection (LOD) and cancer signal origin (CSO) prediction accuracy of various technologies, including WGBS, targeted sequencing, and WGS, and found that WGBS demonstrated the best detection performance [76]. In the second substudy (training and initial validation sets), the study analyzed the methylation sequencing of plasma cfDNA from 2482 cancer patients (covering 50 cancer types) and 4207 healthy individuals, targeting a panel of >100,000 informative methylation regions. Using machine learning, the study developed cancer detection and tissue of origin (TOO) localization capabilities. The results showed a sensitivity of 43.9% and specificity of 99.3% in the validation set, with sensitivity increasing with higher TNM stages and varying across different cancer types. Notably, the TOO prediction accuracy was 93% [77]. In the third substudy (a further validation cohort of 2823 cancer patients and 1254 healthy individuals), the GRAIL test demonstrated a sensitivity of 51.5%, specificity of 99.5%, and CSO prediction accuracy of 88.7% [78]. The CCGA study results highlight the significant potential of the GRAIL test as a multi-cancer early detection (MCED) technology. Its introduction has brought widespread attention to the concept of MCED and accelerated advancements in this field. Beyond CCGA, several other clinical studies are further validating the screening efficacy of the GRAIL test. The STRIVE study (NCT03085888), a large-scale prospective observational study started on 28 February 2017, aims to evaluate the effectiveness of GRAIL’s MCED test in early cancer screening among women. The SUMMIT study (NCT03934866), a prospective observational cohort study that was initiated on 8 April 2019, plans to enroll 13,000 participants aged 55–77 years. Its primary objective is to clinically validate a blood test for the early detection of multiple cancers. However, the results of these two clinical trials have not been published. PATHFINDER (NCT04241796) is another prospective cohort study conducted by GRAIL, aiming to evaluate the real-world decision-making impact of multi-cancer early detection (MCED) testing. This study enrolled 6621 individuals aged 50 and above, including 92 cancer patients. The GRAIL test demonstrated a sensitivity of 38%, specificity of 99.1%, PPV of 38%, and negative predictive value (NPV) of 98.6%. In terms of CSO prediction, the accuracy of the first test was 85%, increasing to 97% when considering the first or second test. Additionally, the study assessed the time to diagnostic resolution (the interval from a physician’s receipt of the test results to diagnostic resolution), with a median time of 79 days (57 days for true positives and 162 days for false positives). This study demonstrated the feasibility of MCED testing in real-world clinical settings, marking a significant step forward in the advancement of GRAIL testing [79]. Further research has evaluated the experimental model of PATHFINDER in different populations. PATHFINDER2 (NCT05155605), a case–control study, is assessing the MCED test in a larger U.S. cohort. This study, initiated on 8 December 2021, plans to enroll 35,885 participants and is expected to complete its primary objectives by 28 February 2026. The University of Oxford’s SYMPLIFY study (ISRCTN10226380) evaluated the effectiveness of MCED testing in individuals with non-specific symptoms (e.g., fatigue, weight loss), with 5461 participants included in the final analysis. The SYMPLIFY study reported a sensitivity of 66.3%, specificity of 98.4%, PPV of 75.5%, NPV of 97.6%, and CSO prediction accuracy of 84.8% [80]. Additionally, the UK’s National Health Service (NHS) Galleri study (ISRCTN 91431511, NCT05611632) is conducting a randomized controlled trial involving 140,000 adults to compare the effectiveness of conventional cancer screening with the MCED test. This study began on 1 July 2021 and is expected to conclude by 28 February 2026. In summary, the GRAIL method, developed based on methylation detection, has demonstrated significant application potential in MCED compared to ctDNA mutation-based detection. With substantial clinical prospects, it represents a promising advancement in early cancer screening.

DNA fragment-based methods exhibit higher sensitivity but lower specificity compared to mutation-based and methylation-based methods. As diagnostic testing requires high sensitivity, a series of studies were prompted to investigate DELFI’s effectiveness in high-risk cancer populations. The DELFI-L101 study evaluated the performance of DELFI-based detection in individuals eligible for routine lung cancer screening, with a planned enrollment of 2660 participants. The study included 958 participants who met the criteria for low-dose CT (LDCT) screening, divided into training and validation sets. Since the target population already required LDCTs, the study adjusted the detection method in the validation set to achieve a clinical sensitivity target of 80%. The results showed an overall sensitivity of 84% and a specificity of 50% in the training set, with similar performance in the validation set (a sensitivity of 84% and a specificity of 53%) [81]. The CASCADE-LUNG study (also known as the DELFI-L201 Study, NCT05306288), another event-driven, multi-site, prospective, observational blood sample collection study, aims to further evaluate the sensitivity and specificity of the DELFI lung cancer screening test in the elevated-risk lung cancer screening population. With a larger target enrollment of 15,000 participants, this study has not yet published the interim results and is expected to complete its primary objectives by 31 March 2025. Thus, the application of the DELFI method remains in its early stages, with no large-scale prospective clinical studies published to date. However, its unique advantages, such as not requiring mutation or methylation detection, lower costs, and high sensitivity, position it as a promising tool for early cancer diagnosis. Future advancements through large-scale clinical trials, technical optimization, and multi-omics integration could establish DELFI as a valuable complement to ctDNA-based early screening and diagnostic approaches.

**Table 3:** Completed and ongoing clinical trials on ctDNA-based early cancer screening test on Clinicaltrial.gov.

Methodology	Purpose	Study	Cancer Type	Total Sample	Conclusion	References/ClinicalTrial.gov Identifier
Mutation detection	Early detection	CancerSEEK	Ovarian, liver, gastric, pancreatic, esophageal, colorectal, lung, and breast cancers	1817	Medium sensitivity: 70% Medium specificity: 99%	[74]
	Multi-cancer early detection	DETECT-A	Multiple cancers	10,006	Medium sensitivity: 27.1% Medium specificity: 98.9%	[75]
	Multi-cancer early detection	ASCEND	Multiple cancers	4620	/	NCT04213326
Methylation detection	Multi-cancer early detection	CCGA	Multiple cancers	4077	Medium sensitivity: 51.5% Medium specificity: 99.5%	[78]
	Multi-cancer early detection	STRIVE	Breast cancer and other invasive cancers, including hematologic malignancies	100,000	/	NCT03085888
	Multi-cancer early detection	SUMMIT	Multiple cancers	13,000	/	NCT03934866
	Multi-cancer early detection	PATHFINDER	Multiple cancers	6621	Medium sensitivity: 38.0% Medium specificity: 99.1%	[79]
	Multi-cancer early detection	PATHFINDER2	Multiple cancers	35,885	/	NCT05155605
	Multi-cancer early detection	SYMPLIFY	Multiple cancers	5461	Medium sensitivity: 66.3% Medium specificity: 98.4%	[80]
	Multi-cancer early detection	NHS-Galleri	Multiple cancers	140,000	/	NCT05611632
	Early detection	K-DETEK	Stomach, esophageal, colorectal, lung, or liver cancer	100,501	Medium sensitivity: 88.0% Medium specificity: 96.0%	[82]
	Multi-cancer early detection	/	Multiple cancers	50,000	/	NCT05673018
	Early detection	/	Lung cancer	600	/	NCT05432128
	Multi-cancer early detection	CADENCE	Multiple cancers	15,000	/	NCT05633342
	Multi-cancer early detection	CHARM2	Hereditary cancer syndromes	1000	/	NCT06726642
	Multi-cancer early detection	ProSight	Lung cancer, colorectal cancer, liver cancer, gastric cancer, and esophageal cancer	2527	/	NCT06790355
	Multi-cancer early detection	/	Gastric cancer	540	/	NCT04511559
	Multi-cancer early detection	/	Lung cancer	300	/	NCT03685669
	Multi-cancer early detection	/	Esophageal squamous cell carcinoma	300	/	NCT03922230
Mutation and methylation detection	Multi-cancer early detection	ASCEND-PANCREATIC	Multiple cancers	7062	/	NCT05556603
	Early detection	/	Lung cancer	900	/	NCT04814407
	Multi-cancer early detection	/	Non-small cell lung cancer	400	/	NCT03301961
Fragment detection	Early detection	DELFI	Breast, colorectal, lung, ovarian, pancreatic, gastric, or bile duct cancer	481	Medium sensitivity: 73.0% Medium specificity: 98.0%	[73]
	Early detection	DELFI-L101	Lung cancer	342	Medium sensitivity: 84.0% Medium specificity: 53.0%	[81]
	Early detection	DELFI-L201	Lung cancer	15,000	/	NCT05306288

### 4.2. Postoperative MRD Detection

The detection of residual tumor cells by flow cytometry has long been recognized as a poor prognostic factor in hematologic malignancies after induction therapy. As a result, MRD (which refers to hematologic tumor cells that persist in the bloodstream)-guided treatment modification is now the standard of care for those non-solid tumors [83,84,85]. Given its well-established role in hematologic cancers, identifying MRD biomarkers in solid tumors could similarly guide personalized adjuvant or consolidation therapy [19].

When it comes to solid tumors, physicians assess the tumor margins for pathological negativity to confirm complete excision during surgery [86], while in postoperative follow-ups, recurrences and metastatic lesions are monitored through imaging examinations [87]. However, in a considerable proportion of patients, a small population of residual tumor cells, known as MRD, may remain undetected due to their presence at levels below the sensitivity of imaging or physical examination [46]. Over time, these cells can proliferate and eventually cause disease recurrence and metastasis [88], whereas ctDNA often has higher sensitivity to identify MRD in solid tumors [19,89]. In MRD ctDNA studies for solid tumors, two main approaches are commonly used: (1) MRD landmark analysis and (2) surveillance analysis. The former assesses the ctDNA status at a specific time point, while the latter involves serial blood sampling over the follow-up period, dynamically monitoring changes in the ctDNA levels. From a clinical perspective, landmark analysis is a single-timepoint, cost-effective test that provides immediate clinical decisions. However, it carries the risk of false negatives, which can be avoided through surveillance analysis [90,91,92].

In predicting postoperative recurrence, Henriksen et al. conducted a systematic study of patients with stage II–III colorectal cancer. By analyzing the postoperative ctDNA in 851 patients, they demonstrated that postoperative ctDNA positivity holds exceptional predictive value for recurrence, achieving a specificity of 98%, sensitivity of 35%, NPV of 89%, and PPV of 75% [93]. Regarding survival prognosis, Everett et al. performed a meta-analysis and found that patients with MRD-positive ctDNA showed significantly higher risks of progression, with hazard ratios (HR) for progression-free survival (PFS) ranging from 3.5 to 43.3 compared to MRD ctDNA-negative patients [19]. According to previous literature reports, the sensitivity of ctDNA for MDR detection varies across studies, but its specificity consistently remains above 80% [92,94,95,96,97,98,99]. Additionally, the ctDNA MRD detected residual disease at a median of 5.8 months earlier than radiographic imaging, highlighting its strong prognostic and therapeutic guidance value [19].

### 4.3. Therapeutic Assessment

In addition to facilitating early recurrence detection, ctDNA enables dynamic, real-time monitoring by repeatedly collecting blood samples from patients and continuously assessing any changes. A sustained decline or disappearance of ctDNA following treatment suggests a favorable therapeutic response, whereas stable or rising ctDNA levels during treatment may indicate resistance or an increased risk of relapse, necessitating timely adjustments to the treatment strategy. Although we previously discussed how MRD ctDNA detection can guide treatment, this section focuses more specifically on the applications of ctDNA in evaluating treatment strategies.

We summarized the international clinical trials from the past five years that evaluated the role of ctDNA in cancer therapeutic assessment, as presented in Table 4. These clinical trials include both solid and non-solid tumors, with the application of ctDNA primarily focusing on the following two aspects: (1) ctDNA alone or integrating ctDNA with other diagnostic tools to develop more effective postoperative prognostic models and stratify the risk of tumor recurrence and survival probability [100,101,102,103,104], and (2) evaluating the efficacy of adjuvant chemotherapy or immunotherapy [100,101,103,104,105,106,107,108]. These studies provide evidence-based guidance for cancer treatment strategies, assisting clinicians in formulating more precise postoperative treatment plans and improving patient survival outcomes.

## 5. Conclusions

In this review, we comprehensively elucidate the biological origins, molecular characteristics, detection methodologies, and clinical implementations of ctDNA, delineating its translational trajectory from fundamental research to clinical practice. Furthermore, we aim to establish a systematic framework for understanding ctDNA’s unique attributes and consolidate robust evidence supporting its clinical utility across diverse oncological contexts. However, research on ctDNA extends far beyond what has been covered here. This study acknowledges limitations in its coverage of clinical research. Although we prioritized the five most prevalent malignancies, they collectively account for less than 50% of the total cancer incidence. The clinical utility of ctDNA in other tumor types requires comprehensive meta-analyses to establish robust evidence. Furthermore, with ongoing technological iterations in detection platforms, such as enhanced precision and standardization of dPCR and NGS, emerging methodologies beyond those discussed herein (e.g., CancerSEEK) are increasingly transitioning into clinical validation. These advancements warrant further investigation and discourse to optimize ctDNA-based diagnostic frameworks. Given its exceptional performance as a minimally invasive and highly sensitive biomarker, we anticipate that, with continued advancements in detection technologies and reductions in cost, ctDNA will become a routine clinical diagnostic tool like conventional blood tests. Its potential extends beyond early cancer screening to real-time disease monitoring, treatment response assessment, and personalized therapeutic decision-making. As ctDNA further integrates into precision oncology, we expect it to significantly enhance cancer diagnostics and patient outcomes, ultimately contributing to improved survival and quality of life.

## Figures and Tables

**Table 1:** Comparisons of ctDNA and cfDNA.

	cfDNA (Cell-Free DNA)	ctDNA (Circulating Tumor DNA)	References
General Description	All DNA fragments	DNA fragments from cancer cells	[22,23,24,25]
Source	Originates from a wide range of cells, including normal, inflammatory, necrotic, and tumor cells	Mainly originates from tumor cells	[22,23,24,25,26,27,28]
Positive Population	Both healthy individuals and patients	Just cancer patients	[29,30,31]
Specificity	Non-specific; does not carry mutations and can derive from various physiological processes	Highly specific; usually carries tumor-related mutations and methylation	/
Length	100 bp–21 kbp	Less than 100 bp	[32,33]
Plasma Concentration			
Healthy Individuals	1–10 ng/mL	Undetectable	[23,34,35,36,37,38,39,40,41]
Cancer Patients	10–1000 ng/mL	0.01–100 ng/mL	
Proportion of Total cfDNA	100% (includes both ctDNA and DNA from normal cells)	<1–10% (can reach up to 40% in some advanced cancers)	[20]
Applications	Prenatal diagnosis, organ transplant monitoring, and detection of inflammatory diseases	Early screening of cancer, tumor profiling, monitoring of treatment resistance, recurrence detection	[19,20,42,43,44,45,46,47,48,49,50]
Clinical Significance	Reflects the overall cellular state in the body and can be used for various disease studies	Reflects tumor burden, mutation status, and treatment response	/

**Table 2:** Comparisons of dPCR and NGS.

	dPCR	NGS	References
Basic Principle	Determines the absolute copy number of target DNA by analyzing endpoint fluorescence signals in micro-reaction units	Reads DNA sequence information using high-throughput sequencing technology	[51,54,57]
Sensitivity	Extremely high, capable of detecting mutation frequencies as low as 0.1% or even lower	Relatively high, capable of detecting low-frequency mutations, but limited by sequencing depth	[56]
Quantification Accuracy	Absolute quantification, independent of standard curves	Relative quantification, dependent on sequencing depth and data normalization	[55,57,59]
Sample Requirement	Low, small amounts of DNA are sufficient for detection	Requires high-quality and relatively large amounts of DNA	[57,58]
Detection Range	Suitable for detecting single or a small number of gene variations	Suitable for large-scale genomic analysis, covering SNPs, Indels, CNVs, and other genetic variations	[51,56,60,61]
Data Analysis	Simple and fast	Requires complex bioinformatics analysis	[63]
Experimental Cost	Low	High	[63]
Clinical Situations Applied	Low-frequency mutations, high-sensitivity quantification scenarios, and limited-sample contexts	Pan-cancer screening with multi-gene panels, tumor heterogeneity profiling through subclonal variant detection, immunotherapy biomarker evaluation, and exploration of unknown resistance mechanisms involving emerging mutations or fusion genes	[51,54,56,57,58,60,61]

**Table 4:** Clinical trials associated with ctDNA in therapeutic assessment.

Cancer Type	Purpose	Stage	Methodology	Sampling Time Points	Total Sample	Conclusion	References
Lung cancer	Determine the efficacy of neoadjuvant chemotherapy plus nivolumab	Stage IIIA	Oncomine tumor mutation load assay	Before and after neoadjuvant treatment (before surgery)	46	ctDNA levels were significantly associated with OS and outperformed radiologic assessments in the prediction of survival and proved the efficacy of neoadjuvant chemotherapy plus nivolumab in resectable NSCLC	[100]
	Establish a ctDNA-based stratification strategy for immunochemotherapy in patients with NSCLC and evaluate its reproducibility and reliability	/	High-throughput panel-based deep-next-generation sequencing and low-pass whole genome sequencing	/	460	Proposed a potential therapeutic algorithm based on the ctDNA-based stratification strategy and shed light on the individualized management of immune–chemotherapies for patients with advanced NSCLC	[101]
Breast Cancer	Predict pCR and risk of metastatic recurrence	Early Stage	WES	At pretreatment (T0); 3 weeks after initiation of paclitaxel (T1); between paclitaxel and anthracycline regimens (T2); or prior to surgery (T3)	84	Personalized monitoring of ctDNA during new adjuvant chemotherapy (NAC) may aid in the real-time assessment of treatment response and help fine-tune a pathologic complete response (pCR) as a surrogate endpoint of survival	[105]
	Examine the predictive and prognostic value of ctDNA	Early Stage	Multiplex PCR	At pretreatment (T0); 3 weeks after the initiation of treatment (T1); at 12 weeks, between paclitaxel-based and anthracycline (AC) regimens (T2); and after NAC before surgery (T3)	283	Maximized and fine-tuned the use of ctDNA as a biomarker of response and survival in patients with high-risk early-stage breast cancer receiving NAC	[106]
	Assess the utility of prospective ctDNA surveillance in TNBC and the activity of pembrolizumab in patients with ctDNA detected [ctDNA positive (ctDNA+)]	Early Stage	dPCR	Three-monthly blood sampling to 12 months (18 months if the samples were missed due to coronavirus disease) after initial therapy	208	Emphasized the importance of commencing ctDNA testing early, with more sensitive and/or frequent ctDNA testing regimes, as well as the activity of pembrolizumab	[107]
Colorectal Cancer	Explore the value of circulating tumor DNA (ctDNA) in combination with MRI in the prediction of pCR before surgery and investigate the utility of ctDNA in risk stratification and prognostic prediction for patients undergoing nCRT and total mesorectal excision (TME)	Advanced Stage	Deep-targeted panel sequencing	At baseline, during nCRT, and after surgery	119	Combining ctDNA and MRI can improve the predictive performance, and combining ctDNA with high-risk features can stratify patients with a high risk of recurrence	[102]
	Assess whether a ctDNA-guided approach could reduce the use of adjuvant chemotherapy without compromising recurrence risk	Stage II	Safe-sequencing system	At week 4 and week 7, after surgery	455	A ctDNA-guided strategy could reduce adjuvant chemotherapy use without increasing the recurrence risk in stage II colon cancer	[103]
Prostate Cancer	Determine the acquired genomic contributors to cross-resistance	Metastatic castration-resistant prostate cancer	Deep-targeted and whole-exome sequencing	At baseline and progression time points	458	The dominant AR genotype continues to evolve during sequential lines of AR inhibition and drives acquired resistance in patients with mCRPC	[108]
Gastric Cancer	Evaluate the predictive value of ctDNA in disease recurrence after adjuvant chemotherapy	Stage II/III	Targeted sequencing panel	Perioperatively and within 3 months after adjuvant chemotherapy	100	Residual ctDNA after ACT effectively predicts high recurrence risk in stage II/III GC, and the combination of tissue-based and circulating tumor features could achieve better risk prediction	[104]
